# Cognitive Biases in the Era of COVID-19 : A Case of *Clostridium sporogenes* Bacteremia in a Patient with Small Bowel Obstruction

**DOI:** 10.1155/2020/8812635

**Published:** 2020-12-05

**Authors:** Marc J. Vecchio, Matthew Jankowich, Hassan Qadir, Melissa Gaitanis, Anupama Menon

**Affiliations:** ^1^Brown University Warren Alpert Medical School, Eddy St, Providence, RI 02903, USA; ^2^Department of Pulmonary and Critical Care Medicine, Providence Veteran Affairs Medical Center, 830 Chalkstone Avenue, Providence, RI 02908, USA; ^3^Department of Internal Medicine, Providence Veteran Affairs Medical Center, 830 Chalkstone Avenue, Providence, RI 02908, USA; ^4^Department of Infectious Diseases, Providence Veteran Affairs Medical Center, 830 Chalkstone Avenue, Providence, RI 02908, USA

## Abstract

*Clostridium sporogenes* bacteremia in immunocompetent patients is rare with very few reported cases in the literature. We present a case of *Clostridium sporogenes* bacteremia in an 81-year-old immunocompetent man with small bowel obstruction and hypoxemia during the COVID-19 pandemic. Routine monitoring of prognostic inflammatory markers for COVID-19 created a unique challenge in the management of our patient who developed sepsis with respiratory symptoms. Upon review, bacteremia from *Clostridium sporogenes* was associated with high mortality rates and could produce similar elevations in the inflammatory markers observed in COVID-19 pneumonia. Further, we reviewed the cognitive biases encountered when monitoring these inflammatory markers during the management of our patient with *Clostridium sporogenes* bacteremia, who was initially thought to have COVID-19 disease. While our patient ultimately tested negative for COVID-19, early administration of empiric antimicrobial therapy without source control failed to prevent clinical decompensation.

## 1. Introduction

*Clostridium sporogenes* is a Gram-positive, anaerobic bacillus that is part of the normal flora found in the human gastrointestinal tract. Although cases of *Clostridium sporogenes* bacteremia have been documented in immunocompromised patients, review of current literature reveals a paucity of reports describing bacteremia in immunocompetent patients. Moreover, none of the most recent reports cite an abdominal source as the nidus of infection. Instead, the most commonly described sources included extensive wound tissue necrosis, skin ulceration, and osteomyelitis [1, 2]. Additionally, no reports outline the management of this rare bacteremia or the challenges of navigating cognitive biases during the intercurrent SARS-CoV-2 (COVID-19) pandemic.

## 2. Case Report

An 81-year-old man with a medical history of chronic kidney disease stage III, obesity with a body mass index of 52.0, cholecystectomy, and appendectomy presented in April 2020 from an assisted living facility with two weeks of lower abdominal pain, anorexia, and abdominal distention. He also reported at least 3 days of progressive dyspnea and the absence of bowel movements. Upon presentation to the hospital, he required 2 L/min oxygen by nasal cannula, but was afebrile and hemodynamically stable. Physical examination revealed abdominal distention with tenderness to palpation, voluntary guarding, and an umbilical hernia.

**Figure 1:**
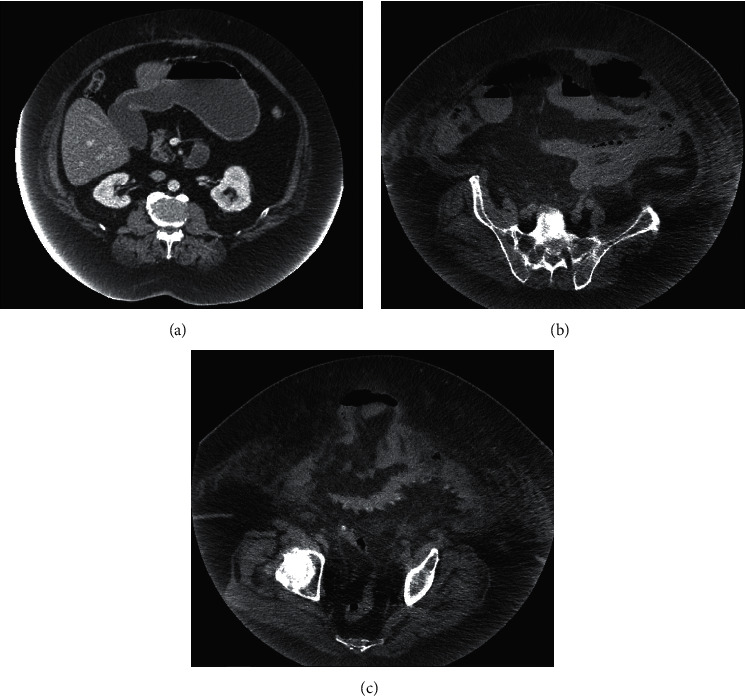
Dilated stomach (a) and small bowel loops throughout the abdomen with a fat-containing ventral wall hernia (b). Umbilical hernia containing fat and distal small bowel loops, transition point (c).

Admission laboratory testing revealed a white blood cell count of 25.7 K/cmm, sodium of 133 mmol/L, chloride of 95 mmol/L, bicarbonate of 28 mmol/L, creatinine of 1.0 mg/dL, total bilirubin of 2.0 mg/dL with normal transaminase levels, lactate dehydrogenase of 186 units/L, lactate of 4.1 mmol/L, procalcitonin of 0.15 ng/mL, ferritin of 143.87 ng/mL, and high-sensitivity C-reactive protein of 23.0 mg/L. Respiratory pathogen panel was negative, and SARS-CoV-2 was not detected by reverse transcriptase-polymerase chain reaction (RT-PCR) on nasopharyngeal swab. Two sets of blood cultures and one urine culture were obtained. Computed tomography of the abdomen and pelvis with intravenous contrast showed a distended stomach, dilated loops of small bowel, a fat-containing ventral wall hernia, and an umbilical hernia containing small bowel that was concerning for a transition point (Figure 1). A nasogastric tube was placed by the general surgery team in the emergency department with immediate return of 2 liters of feculent material. The patient's umbilical hernia was manually reduced with palliation of the abdominal pain. The patient also received 3 liters of crystalloid fluid with improvement in both lactate and white blood cell count.

**Figure 2:**
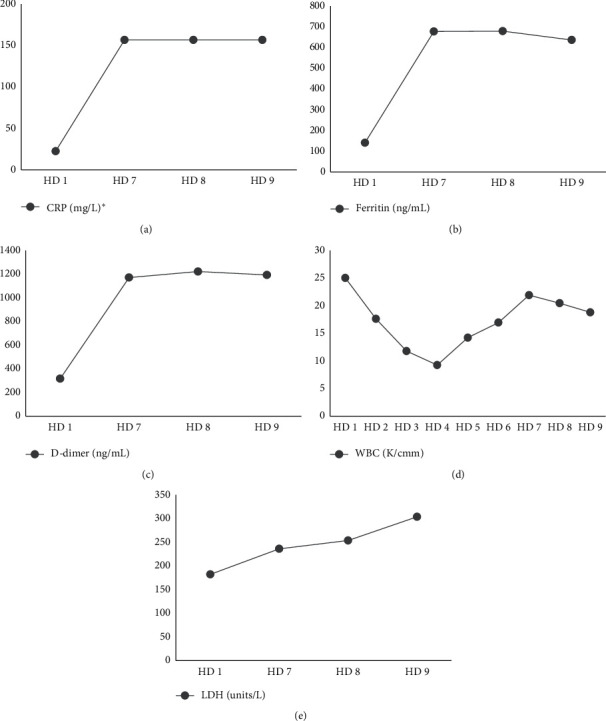
A graphical representation of (a) serum C-reactive protein, (b) serum ferritin, (c) quantitative D-dimer, (d) white blood cell count, and (e) serum lactate dehydrogenase. HD, hospital day. ^*∗*^Unstable to quantify CRP value greater than 160 mg/L.

After the initial improvement in exam and inflammatory markers with nasogastric decompression and crystalloids, the patient was admitted to the general surgery service for conservative management of suspected small bowel obstruction. Over the next 4 hospital days, the patient had decreased abdominal pain, was stable on room air, and had resolution of all laboratory abnormalities. His initial blood cultures on admission remained negative. On hospital day 5, the patient's nasogastric tube was removed without complication after he began passing flatus. Several hours later, he became hypoxemic to 88% on room air with increased abdominal distension, hypotension, and tachycardia. Empiric vancomycin, piperacillin-tazobactam, doxycycline, and crystalloids were instituted after repeat blood and urine culture collection. Portable upright abdominal radiograph showed distended loops of bowel without free air, while chest radiograph showed new patchy bibasilar opacities. Evaluation by the surgical team revealed a stable abdominal exam without rebound tenderness following resuscitation and antibiotics. Surgical bowel exploration was considered, but deferred due to his stable abdominal exam and high intraoperative mortality risk. He remained afebrile, but had increasing tachypnea and required up to 4 L/min oxygen by nasal cannula. Further imaging studies were deferred at the time due to the patient's tenuous respiratory status. Laboratory studies revealed a white blood cell count increase to 14.6 k/cmm, creatinine of 2.5 mg/dL, ferritin of 691.79 ng/dL, procalcitonin level of 15.93 ng/mL, D-dimer of 1203 ng/mL, lactate dehydrogenase of 241 units/L, high-sensitivity C-reactive protein of greater than 160 mg/L, and lactate of 3.1 mmol/L (Figure 2). COVID-19 pneumonia was suspected, and a repeat SARS-CoV-2 RT-PCR test was sent and was negative. Given concerns for a false-negative RT-PCR test result, the patient was transferred to the dedicated COVID-19 unit for airborne isolation in a negative pressure room. The following day, the patient's inflammatory markers worsened with persistent hypoxemia. A third nasopharyngeal swab was sent for RT-PCR, but was negative. On hospital day 7, both sets of repeat blood cultures grew a Gram-positive bacillus later identified as *Clostridium sporogenes*. Sensitivities were not performed as per the microbiology laboratory's protocol for anaerobic organisms, but the isolate was beta-lactamase negative.

After stabilization, repeat computed tomography of the chest, abdomen, and pelvis without intravenous contrast revealed focal ground glass opacities in the left upper lobe, bibasilar airspace disease, and new asymmetric thickening of the left rectus muscle with fluid consistent with abscess formation. The patient's creatinine peaked at 4.5 mg/dL before decreasing with judicious administration of intravenous crystalloid for suspected acute tubular necrosis. Urine culture showed no growth, and urine legionella antigen was not detected. The patient was treated with piperacillin-tazobactam and later meropenem with improvement of most inflammatory markers, but with persistent leukocytosis. On hospital day 10, after discussion with the surgery service regarding surgical intervention and the associated mortality risk, the patient decided to pursue comfort measures only. He was transferred to an inpatient hospice facility and died two days later.

## 3. Discussion

Bacteremia due to the anaerobic genus *Clostridium* comprises approximately 2.0% of all isolates reported in several literature reviews [3]. A recent retrospective cohort conducted from 2010 to 2018 examined the clinical relevance of *Clostridium* bacteremia by reviewing 688,189 blood cultures. Only 81 patients grew more than one blood culture for *Clostridium* species, with 4.9% growing *Clostridium sporogenes*. Further, few patients in this group were immunocompetent, with the most common comorbidities being cancer, chemotherapy, and recent gastrointestinal surgery. The most commonly reported infectious sources were from the gastrointestinal tract, skin, and soft tissue [3, 4]. *Clostridium* species growth in blood cultures is rarely considered a contaminant and should prompt immediate treatment. In a study comparing *Bacillus* with *Clostridium* species bacteremia, mortality rates of *Clostridium* species were higher than those of *Bacillus* species, but approached rates seen in other patients with bacteremia [5]. In cases of clinically relevant *Clostridium* bacteremia, the 30-day mortality was 31.4%, with early empiric antimicrobial therapy significantly improving survival rates [4]. While the exact mechanism of pathogenicity from *Clostridium sporogenes* is unclear, antibiotics such as penicillin and clindamycin may overcome resistance and toxin formation [6]. However, the initiation of broad-spectrum antimicrobial therapy is often the best initial treatment modality as *Clostridium* bacteremia is frequently polymicrobial in nature [5].

While our patient did not have COVID-19 infection, it is interesting to note his inflammatory markers emulated what has been encountered in patients with severe COVID-19 disease. Our case illustrates several challenging cognitive biases clinicians will likely encounter during the COVID-19 pandemic. Recognizing and navigating these cognitive biases are crucial to preventing delay in diagnosis of another significant medical condition. For example, as a result of our patient's worsening hypoxemia and benign abdominal exam, three tests for COVID-19 were obtained, thus illustrating confirmation bias in which reinforcement of preconceived notions is favored over contradictory information [7]. In addition, availability bias increased the suspicion for COVID-19 infection due to the observed acute elevation in high-sensitivity C-reactive protein (hsCRP), lactate dehydrogenase (LDH), ferritin, D-dimer, and procalcitonin. Lastly, it was essential to avoid anchoring on a diagnosis of COVID-19 with the delay in blood culture positivity. Notably, further strengthening these biases was the perceived high exposure risk to the house staff participating in the care of this patient. With the healthcare system facing this unprecedented crisis, clinicians should strive to acknowledge and understand the nature of these biases in an effort to determine how best to treat patients.

Elevated inflammatory markers, such as IL-6, hsCRP, LDH, ferritin, D-dimer, and procalcitonin, have been observed more frequently in severe cases of COVID-19 compared to non‐severe cases [8–13]. Terpos, E., et al. found elevation in these markers resulted in higher likelihood of intensive care unit admission, acute respiratory distress syndrome, mechanical ventilation, severe infection, and death [13]. However, it is worth emphasizing these inflammatory markers remain nonspecific, and a high suspicion for COVID-19 should not delay empiric antibacterial treatment if a patient's symptoms and laboratory results could also result from a bacterial infection. Indeed, biomarkers such as CRP, ferritin, and procalcitonin are frequently elevated in generalized inflammatory conditions including bacterial sepsis. Substantial elevations in these markers increase the sensitivity for a diagnosis of infection, but are nonspecific for a source of infection as illustrated in our patient with *Clostridium sporogenes* bacteremia who did not have COVID-19 [14, 15].

In conclusion, this case outlines the challenges of managing a rare case of *Clostridium sporogenes* bacteremia from small bowel obstruction in an immunocompetent adult during the COVID-19 pandemic. More importantly, it emphasizes the need for clinicians to preserve clinical acumen and be mindful of cognitive biases when evaluating patients with clinical and laboratory parameters suggestive of COVID-19 who ultimately prove to have alternative diagnoses.

## References

[1] Abusnina W., Shehata M., Karem E., Koc Z., Khalil E. (2019). *Clostridium sporogenes* bacteremia in an immunocompetent patient. *IDCases*.

[2] Alataby H. A., Krishnamoorthy V., Ndzelen L. (2020). *Clostridium sporogenes* causing bacteremia originated from the skin and soft tissue infection in an immunocompetent patient - case report and literature review. *International Journal of Critical Care and Emergency MedicinE*.

[3] Rechner P. M., Agger W. A., Mruz K., Cogbill T. H. (2001). Clinical features of clostridial bacteremia: a review from a rural area. *Clinical Infectious Diseases*.

[4] Stabler S., Titécat M., Duployez C. (2020). Clinical relevance of *Clostridium* bacteremia: an 8-year retrospective study. *Anaerobe*.

[5] Benjamin B., Kan M., Schwartz D., Siegman-Igra Y. (2006). The possible significance of *Clostridium* spp. in blood cultures. *Clinical Microbiology and Infection*.

[6] Hara-Kudo Y., Ogura A., Noguchi Y., Kumagai S. (1997). Characteristics of toxicity and haemorrhagic toxin produced by *Clostridium sporogenes* in various animals and cultured cells. *Journal of Medical Microbiology*.

[7] Zagury-Orly I., Schwartzstein R. M. (2020). Covid-19 - a reminder to reason. *The New England Journal of Medicine*.

[8] McCreary E. K., Pogue J. M. (2020). Coronavirus disease 2019 treatment: a review of early and emerging options. *Open Forum Infectious Diseases*.

[9] Chen T., Wu D., Chen H. (2020). Clinical characteristics of 113 deceased patients with coronavirus disease 2019: retrospective study. *BMJ*.

[10] Liu Y., Yang Y., Zhang C. (2020). Clinical and biochemical indexes from 2019-nCoV infected patients linked to viral loads and lung injury. *Science China Life Sciences*.

[11] Ruan Q., Yang K., Wang W., Jiang L., Song J. (2020). Correction to: Clinical predictors of mortality due to COVID-19 based on an analysis of data of 150 patients from Wuhan, China. *Intensive Care Medicine*.

[12] Wang Z., Yang B., Li Q., Wen L., Zhang R. (2020). Clinical features of 69 cases with coronavirus disease 2019 in Wuhan, China. *Clinical Infectious Diseases*.

[13] Terpos E., Ntanasis-Stathopoulos I., Elalamy I. (2020). Hematological findings and complications of COVID-19. *American Journal of Hematology*.

[14] Sankar V., Webster N. R. (2013). Clinical application of sepsis biomarkers. *Journal of Anesthesia*.

[15] Missano Florido M., Assunção M., Mazza B. (2012). Evaluation of iron, transferrin and ferritin serum levels in patients with severe sepsis and septic shock. *Critical Care*.

